# Supplementary material for: Systematic Review of Peer-Reviewed Literature on Global Condom Promotion Programs
Source: Int J Environ Res Public Health. 2020 Mar 27;17(7):2262. doi: 10.3390/ijerph17072262 (PMC7177514; doi:10.3390/ijerph17072262)
Supplement: Supplementary file 1 [file ijerph-17-02262-s001.pdf]

Table S1. Summary of Articles Reviewed

Citation	Populations	Region	Product	4 P(s) of Marketing	Intervention Components	Design	Significant Effects
Chapman, S., Jafa, K., Longfield, K., Vielot, N., Buszin, J., Ngamkitpaiboon, L., et al. (2012). Condom social marketing in Sub-Saharan Africa and the total market approach. Sexual Health, 9(1), 44-50.	Adolescents	Sub-Saharan Africa	Male Condom	Product Price Place Promotion	Mass media Community Outreach	Observational	Product/Behavior Awareness Pre-behavioral Behavioral Sales and Distribution
Evans, W. D., Taruberekera, N., Longfield, K., & Snider, J. (2011). Brand equity and willingness to pay for condoms in Zimbabwe. Reproductive Health, 8(1)	General Populations	Sub-Saharan Africa	Male Condom	Product Price Place Promotion	Mass media	Experimental	Product/Behavior Awareness Pre-behavioral Behavioral Sales and Distribution
Adams, J., Neville, S., Parker, K., & Huckle, T. (2017). Influencing condom use by gay and bisexual men for anal sex through social marketing: A program evaluation of get it on! Social Marketing Quarterly, 23(1), 3-17.	Adolescents Young Adult LGBTQ	Oceania	Male Condom	Product Price Place Promotion	Mass media Community Outreach	Quasi-Experimental	Pre-behavioral Behavioral
Agha, S. (2000). An evaluation of adolescent sexual health programs in Cameroon, Botswana, South Africa, and Guinea. Washington, D.C., Population	Adolescents	Sub-Saharan Africa	Male Condom	Product Price Place Promotion	Mass media Community Outreach	Observational	Pre-behavioral Behavioral

Citation	Populations	Region	Product	4 P(s) of Marketing	Intervention Components	Design	Significant Effects
Services International [PSI], Research Division, 2000.							
Agha, S. (2001). Patterns of use of the female condom after one year of mass marketing. AIDS Education and Prevention, 13(1), 55-64.	Women Men Other	Sub-Saharan Africa	Both Male and Female Condom	Product Price Place Promotion	Mass media Community Outreach	Experimental	Product/Behavior Awareness Pre-behavioral Behavioral
Agha, S. (2001). Intention to use the female condom following a mass-marketing campaign in Lusaka, Zambia. American Journal of Public Health, 91(2), 307-310.	Women Men Other	Sub-Saharan Africa	Both Male and Female Condom	Product Price Place Promotion	Mass media Community Outreach	Experimental	Product/Behavior Awareness Pre-behavioral Behavioral
Agha, S. (2001). The impact of the Kenya social marketing program on personal risk perception, perceived self-efficacy and on other behavioral predictors. Washington, D.C., Population Services International PSI], Research Division, 2001.	General Populations Other	Sub-Saharan Africa	Male Condom	Product Price Place Promotion	Mass media Community Outreach	Quasi-Experimental	Product/Behavior Awareness Pre-behavioral Behavioral
Agha, S. (2002). A quasi-experimental study to assess the impact of four adolescent sexual health interventions in Sub-Saharan Africa. International Family Planning Perspectives, 28(2), 67-70.	Adolescents Women Men	Sub-Saharan Africa	Male Condom	Product Price Place Promotion	Mass media Community Outreach	Quasi-Experimental	Product/Behavior Awareness Pre-behavioral

Citation	Populations	Region	Product	4 P(s) of Marketing	Intervention Components	Design	Significant Effects
Agha, S., & Van Rossem, R. (2002). Impact of mass media campaigns on intentions to use the female condom in Tanzania. International Family Planning Perspectives, 28(3), 151-158.	General Populations	Sub-Saharan Africa	Female Condom	Product Price Place Promotion	Mass media Community Outreach Health Care Provider	Quasi-Experimental	Product/Behavior Awareness Pre-behavioral
Agha, S. (2003). The impact of a mass media campaign on personal risk perception, perceived self-efficacy and on other behavioural predictors. AIDS Care, 15(6), 749-762.	General Populations	Sub-Saharan Africa	Male Condom	Product Price Place Promotion	Mass media Community Outreach	Quasi-Experimental	Product/Behavior Awareness Pre-behavioral Behavioral
Agha, S., Karlyn, A., & Meekers, D. (2001). The promotion of condom use in non-regular sexual partnerships in urban Mozambique. Health Policy and Planning, 16(2), 144-151.	General Populations Other	Sub-Saharan Africa	Male Condom	Product Price Place Promotion	Mass media Community Outreach Community Mobilization	Quasi-Experimental	Product/Behavior Awareness Pre-behavioral Behavioral
Agha, S., & Meekers, D. (2010). Impact of an advertising campaign on condom use in urban Pakistan. Studies in Family Planning, 41(4), 277-290.	General Populations	Indian subcontinent	Male Condom	Product Price Place Promotion	Mass media	Observational	Product/Behavior Awareness Pre-behavioral Behavioral
Agha, S., & Beaudoin, C. E. (2012). Assessing a thematic condom advertising campaign on condom use in urban Pakistan. Journal of Health Communication, 17(5), 601-623.	General Populations	Indian subcontinent	Male Condom	Product Price Place Promotion	Mass media	Observational	Product/Behavior Awareness Pre-

Citation	Populations	Region	Product	4 P(s) of Marketing	Intervention Components	Design	Significant Effects
							behavioral Behavioral
Artz, L., Macaluso, M., Kelaghan, J., Austin, H., Fleenor, M., Robey, L., et al. (2005). An intervention to promote the female condom to sexually transmitted disease clinic patients. Behavior Modification, 29(2), 318-369.	Adolescents Young Adult Women Urban Minority	US	Female Condom	Product Price Place Promotion	Mass media	Observational	Behavioral
Ashraf, N., Bandiera, O., & Jack, B. K. (2014). No margin, no mission? A field experiment on incentives for public service delivery. Journal of Public Economics, 120, 1-17.	Women Men Rural Urban	Sub-Saharan Africa	Male Condom	Product Price Place Promotion	Community Outreach	Experimental	Product/Behavior Awareness Pre-behavioral Behavioral Sales and Distribution
Aung, T., Thet, M. M., Sudhinaraset, M., & Diamond-Smith, N. (2019). Impact of a social franchise intervention program on the adoption of long and short acting family planning methods in hard to reach communities in Myanmar. Journal of Public Health (Oxford, England), 41(1),	Women	South East Asia	Both Male and Female Condom	Product Price Place Promotion	Community Outreach Health Care Provider	Observational	Product/Behavior Awareness Pre-behavioral Behavioral Sales and Distribution

Citation	Populations	Region	Product	4 P(s) of Marketing	Intervention Components	Design	Significant Effects
Basu, I., Jana, S., Rotheram-Borus, M., Swendeman, D., Lee, S., Newman, P., et al. (2004). HIV prevention among sex workers in india. Jaids-Journal of Acquired Immune Deficiency Syndromes}, 36(3), 845-852.	Sex Workers	India	Male Condom	Product Place Promotion	Community Outreach Health Care Provider	Experimental	Product/Behavior Awareness Pre-behavioral Behavioral
Benzaken, A. S., Garcia, E. G., Gomes Sardinha, J. C., Pedrosa, V. L., & Paiva, V. (2007). Community-based intervention to control STD/AIDS in the Amazon region, brazil. Revista De Saude Publica}, 41(2)	Women Men Sex Workers Rural Indigenious	Latin America	Male Condom	Product Price Place Promotion	Community Mobilization	Observational	
Bhaskar, R. S. (2012). Impact of a behaviour change intervention model for promotion of condoms amongst young adult males in an occupational setting. Medical Journal Armed Forces India, 68(4), 316-321.	General Populations	India	Male Condom		Health Care Provider	Experimental	Pre-behavioral
Brent, R. (2009). A cost-benefit analysis of a condom social marketing programme in Tanzania. Applied Economics}, 41(4), 497-509.	General Populations	Sub-Saharan Africa	Male Condom	Product Price Place Promotion	Mass media	Observational	Behavioral Sales and Distribution
Brown, B. (2000). At Nicaraguan motels rented for sexual encounters, making condoms available in rooms increases use. International Family Planning Perspectives, 26(4), 201-202.	General Populations	Latin America	Male Condom	Place Promotion	Mass media Social Media	Observational	Sales and Distribution

Citation	Populations	Region	Product	4 P(s) of Marketing	Intervention Components	Design	Significant Effects
Bull, S. S., Cohen, J., Ortiz, C., & Evans, T. (2002). The POWER campaign for promotion of female and male condoms: Audience research and campaign development. Health Communication, 14(4), 475-491.	Adolescents Women Urban	US	Both Male and Female Condom	Product Price Place Promotion	Not Reported	Observational	
Bull, S. S., Posner, S. F., Ortiz, C., Beaty, B., Benton, K., Lin, L., et al. (2008). POWER for reproductive health: Results from a social marketing campaign promoting female and male condoms. Journal of Adolescent Health}, 43}(1), 71-78.	Adolescents Women Urban	US	Both Male and Female Condom	Product Price Place Promotion	Not Reported	Quasi-Experimental	Behavioral
Bull, S. S., Levine, D. K., Black, S. R., Schmiege, S. J., & Santelli, J. (2012). Social media-delivered sexual health intervention: A cluster randomized controlled trial. American Journal of Preventive Medicine, 43(5), 467-474.	Adolescents Women Men Urban	US	Male Condom	Product Price Place Promotion	Social Media	Experimental	Behavioral
Bulsara, A. L. (2000). Social marketing for adolescent sexual health: Results of operations research projects in Botswana, Cameroon, Guinea, and South Africa Washington, D.C., Population Services International PSI], 2000 Jun.	Adolescents	Sub-Saharan Africa	Male Condom	Product Price Place Promotion	Mass media Community Outreach	Observational	Pre-behavioral Behavioral
Burke, R. C., Wilson, J., Bernstein, K. T., Grosskopf, N., Murrill, C., Cutler, B., et al. (2009). The NYC condom: Use and acceptability of New York City's branded	General Populations	US	Male Condom	Product Price Place Promotion	Community Outreach	Observational	Product/Behavior Awareness Pre-

Citation	Populations	Region	Product	4 P(s) of Marketing	Intervention Components	Design	Significant Effects
condom. American Journal of Public Health, 99(12)					Community Mobilization		behavioral Behavioral
Burke, R. C., Wilson, J., Kowalski, A., Murrill, C., Cutler, B., Sweeney, M., et al. (2011). NYC condom use and satisfaction and demand for alternative condom products in new york city sexually transmitted disease clinics. Journal of Urban Health-Bulletin of the New York Academy of Medicine}, 88}(4), 749-758.	General Populations	US	Male Condom	Product Price Place Promotion	Health Care Provider	Observational	Product/Behavior Awareness Pre-behavioral Behavioral
CerdeÃ±o, A. F., MartÃnez-Donate, A. P., Zellner, J. A., SaÃ±udo, F., Carrillo, H., Engelberg, M., et al. (2012). Marketing HIV prevention for heterosexually identified latino men who have sex with men and women: The hombres sanos campaign. Journal of Health Communication, 17(6), 641-658.	LGBTQ	Latin America	Male Condom	Product Price Place Promotion	Community Outreach	Observational	
Cheng, H. D. (2012). Promoting contraceptive use more effectively among unmarried male migrants in construction sites in China: A pilot intervention trial. Asia - Pacific Journal of Public Health, 24(5), 806-815.	General Populations	East Asia	Male Condom	Product Place Promotion	Mass mediaOther	Quasi-Experimental	Product/Behavior Awareness Pre-behavioral Behavioral
Chiang, K. -., Chan, A., & Milan, R. (2018). Social marketing and advertising appeals: On perception and intention to purchase condoms among college	Adolescents	US	Male Condom	Product Promotion	Mass media	Experimental	Pre-behavioral Behavioral

Citation	Populations	Region	Product	4 P(s) of Marketing	Intervention Components	Design	Significant Effects
students. International Journal of Healthcare Management, 11(2), 71-78.							
Crosby, R., DiClemente, R. J., Charnigo, R., Snow, G., & Troutman, A. (2009). A brief, clinic-based, safer sex intervention for heterosexual African american men newly diagnosed with an STD: A randomized controlled trial. American Journal of Public Health, 99 Suppl. 1, S96-103	Adolescents Young Adult Men Minority	US	Male Condom	Product Place Promotion	Health Care Provider	Experimental	Pre-behavioral Behavioral
Darden, C. (2006). Promoting condoms in brazil to men who have sex with men. Reproductive Health Matters, 14(28),	LGBTQ	Latin America	Male Condom	Product Price Place Promotion	Mass media	Experimental	
Davis, K. C., Uhrig, J., Bann, C., Rupert, D., & Fraze, J. (2011). Exploring African American women's perceptions of a social marketing campaign to promote HIV testing. Social Marketing Quarterly, 17(3), 39-60.	Adolescents Young Adult Women Minority	US	Male Condom	Product Price Place Promotion	Mass media	Experimental	Product/Behavior Awareness Pre-behavioral Behavioral
Des Jarlais, D. C., McKnight, C., Arasteh, K., Feelemyer, J., Perlman, D., Hagan, H., et al. (2014). Use of the "NYC condom" among people who use drugs. Journal of Urban Health, 91(3), 547-554.	Urban Other	US	Male Condom	Product Price Place Promotion	Mass media	Observational	Product/Behavior Awareness Pre-behavioral Behavioral Sales and Distribution

Citation	Populations	Region	Product	4 P(s) of Marketing	Intervention Components	Design	Significant Effects
Des Jarlais, D. C., Arasteh, K., McKnight, C., Feelemyer, J., Hagan, H., Cooper, H. L. F., et al. (2014). Combined HIV prevention, the New York city condom distribution program, and the evolution of safer sex behavior among persons who inject drugs in new york city. AIDS and Behavior, 18(3), 443-451.	Urban Other	US	Male Condom	Product Place Promotion	Mass media	Observational	Product/Behavior Awareness Pre-behavioral Behavioral
Donate, A. P. M., Zellner, J. A., Sanudo, F., Fernandez-Cerdeno, A., Hovell, M. F., Sipan, C. L., et al. (2010). Hombres sanos: Evaluation of a social marketing campaign for heterosexually identified Latino men who have sex with men and women. American Journal of Public Health}, 100}(12), 2532-2540.	LGBTQ Minority	US	Male Condom		Mass media Community Outreach	Observational	Product/Behavior Awareness Pre-behavioral Behavioral
Drake, J. K., Thi Thanh, L. H., Suraratdecha, C., Thi Thu, H. P., & Vail, J. G. (2010). Stakeholder perceptions of a total market approach to family planning in Viet Nam. Reproductive Health Matters, 18(36), 46-55.	Adolescents Young Adult	South East Asia	Male Condom	Product Price Place Promotion	Mass media	Observational	
Drake, J. K., Espinoza, H., Suraratdecha, C., Lacayo, Y., Keith, B. M., & Vail, J. G. (2011). Stakeholder perceptions of a total market approach to family planning in Nicaragua. Revista Panamericana De Salud Publica/Pan American Journal of Public Health, 29(5), 329-336.	Adolescents Young Adult	Latin America	Male Condom	Product Price Place Promotion	Mass media	Observational	

**Citation**	**Populations**	**Region**	**Product**	**4 P(s) of Marketing**	**Intervention Components**	**Design**	**Significant Effects**
Eloundou-Enyegue, P., Meekers, D., & CalvÃ¨s, A. E. (2005). From awareness to adoption: The effect of aids education and condom social marketing on condom use in Tanzania (1993-1996). Journal of Biosocial Science, 37(3), 257-268.	General Populations	Sub-Saharan Africa	Male Condom	Product Price Place Promotion	Mass media	Observational	Product/Behavior Awareness Pre-behavioral Behavioral
Evans, W. D., Longfield, K., Shekhar, N., Rabemanatsoa, A., Reerink, I., & Snider, J. (2012). Social marketing and condom promotion in Madagascar: A case study in brand equity research. Obregon/The handbook of global health communication (pp. 330-347)	General Populations	Sub-Saharan Africa	Male Condom	Product Price Place Promotion	Mass media	Observational	
Farris, K. B., Aquilino, M. L., Batra, P., Marshall, V., & Losch, M. E. (2015). Impact of a passive social marketing intervention in community pharmacies on oral contraceptive and condom sales: A quasi-experimental study. BMC Public Health, 15(1)	General Populations	US	Male Condom		Health Care Provider	Quasi-Experimental	Sales and Distribution
Flowers, P., McDaid, L. M., & Knussen, C. (2013). Exposure and impact of a mass media campaign targeting sexual health amongst Scottish men who have sex with men: An outcome evaluation. BMC Public Health, 13	LGBTQ Men	Western Europe/ Canada/	Male Condom	Product Price Place Promotion		Quasi-Experimental	Product/Behavior Awareness Pre-behavioral Behavioral

Citation	Populations	Region	Product	4 P(s) of Marketing	Intervention Components	Design	Significant Effects
Fontu, A. (2014). Exploring the impact of social marketing of female condoms in the city of Kumba, Cameroon. 2010-2011. Aids Research and Human Retroviruses}, 30(1), A199-A200.	Women	Sub-Saharan Africa	Female Condom	Product Place Promotion	Community Outreach	Observational	Product/Behavior Awareness
Geibel, S., King'ola, N., Temmerman, M., & Luchters, S. (2012). The impact of peer outreach on HIV knowledge and prevention behaviours of male sex workers in Mombasa, Kenya. Sexually Transmitted Infections}, 88}(5), 357-362}.	LGBTQ Men Sex Workers	Sub-Saharan Africa	Male Condom	Promotion	Community Outreach	Observational	Pre-behavioral Behavioral
Ganesan, R. (2017). Assessment of the retail environment of male condoms in Kenya, Nigeria, South Africa, Zambia, and Zimbabwe Arlington, Virginia, JSI Research and Training Institute, Strengthening High Impact Interventions for an AIDS-free Generation AIDS Free] 2017.	General Populations	Sub-Saharan Africa	Male Condom	Price Place Promotion	Not Reported	Observational	Behavioral Sales and Distribution
Grey, M. N. (2010). Central American sex workers' introduction of the female condom to different types of sexual partners. AIDS Education and Prevention, 22(5), 466-481.	Sex Workers	Latin America	Female Condom		Other	Observational	Behavioral

Citation	Populations	Region	Product	4 P(s) of Marketing	Intervention Components	Design	Significant Effects
Harris, A. O., Jubwe, S., Kennedy, S. B., Taylor, C. H., Martin, R. B., Bee, E. M., et al. (2011). Condom social marketing program to prevent HIV/AIDS in post conflict Liberia. African Health Sciences, 11, S81.	General Populations	Sub-Saharan Africa	Both Male and Female Condom	Place Promotion	Community Outreach Community Mobilization Health Care Provider	Observational	Sales and Distribution
Hernandez, J. H., Akilimali, P. Z., Muanda, M. F., Glover, A. L., & Bertrand, J. T. (2018). Evolution of a large-scale community-based contraceptive distribution program in Kinshasa, DRC based on process evaluation. Global Health Science and Practice, 6(4), 657-667.	Women Health Care providers Influencers	Sub-Saharan Africa	Male Condom	Product Place Promotion	Community Mobilization Health Care Provider	Observational	Sales and Distribution
Hill, C. A., & Abraham, C. (2008). School-based, randomised controlled trial of an evidence-based condom promotion leaflet. Psychology and Health, 23(1), 41-56.	Adolescents	Western Europe/ Canada /	Male Condom	Promotion	Other	Experimental	Pre-behavioral Behavioral
Hoke, T. H., Feldblum, P. J., Van Damme, K., Nasution, M. D., Grey, T. W., Wong, E. L., et al. (2007). Randomised controlled trial of alternative male and female condom promotion strategies targeting sex workers in Madagascar. Sexually Transmitted Infections, 83(6), 448-453.	Women Sex Workers	Sub-Saharan Africa	Both Male and Female Condom	Product Promotion	Community Outreach Health Care Provider	Experimental	Behavioral
Joanis, C., Beksinska, M., Hart, C., Tweedy, K., Linda, J., & Smit, J. (2011). Three new female condoms: Which do	Women	Sub-Saharan Africa	Female Condom	Product	Not Reported	Experimental	Product/Behavior Awareness

Citation	Populations	Region	Product	4 P(s) of Marketing	Intervention Components	Design	Significant Effects
South-African women prefer? Contraception}, 83}(3), 248-254}.							
Katende, B. R. (2004). Multi-media campaign exposure effects on knowledge and use of condoms for STI and HIV / AIDS prevention in Uganda. Evaluation and Program Planning, 27, 397-407.	Women Men	Sub-Saharan Africa	Male Condom	Promotion	Mass media	Experimental	Pre-behavioral Behavioral
Kennedy, M. G., Mizuno, Y., Seals, B. F., Myllyluoma, J., & Weeks-Norton, K. (2000). Increasing condom use among adolescents with coalition-based social marketing. Aids, 14(12), 1809-1818. doi:10.1097/00002030-200008180-00017	Adolescents	US	Male Condom	Product Price Place Promotion	Mass media Community Outreach Community Mobilization Other	Observational	Pre-behavioral Behavioral
Kennedy, S. B., Nolen, S., Pan, Z., Smith, B., Applewhite, J., & Vanderhoff, K. J. (2013). Effectiveness of a brief condom promotion program in reducing risky sexual behaviours among African American men. Journal of Evaluation in Clinical Practice, 19(2), 408-413.	Adolescents Men	US	Male Condom	Promotion	Community Outreach Health Care Provider	Experimental	Product/Behavior Awareness Pre-behavioral Behavioral
Liao, N. L. (2013). Promoting female condoms in the sex industry in 4 towns of southern china: Context matters. Sexually Transmitted Diseases, 40(3), 264-270.	Women Sex Workers Rural Urban	East Asia	Female Condom	Product Place Promotion	Community Outreach Health Care Provider	Observational	Product/Behavior Awareness Behavioral
Lin, Y. J., Lee, C. H., Chang, C. C., & Lin, C. H. (2016). Evaluation of a video-based intervention to promote condom use among college students in Taiwan. Studies	Adolescents Young Adult	East Asia	Male Condom	Promotion		Observational	Pre-behavioral

Citation	Populations	Region	Product	4 P(s) of Marketing	Intervention Components	Design	Significant Effects
in Health Technology and Informatics, 226, 101-104.							
Longfield, K., Panyanouvong, X., Chen, J., & Kays, M. B. (2011). Increasing safer sexual behavior among Lao Kathoy through an integrated social marketing approach. BMC Public Health,	Adolescents Young Adult LGBTQ Women	South East Asia	Male Condom	Product Price Place Promotion	Community Outreach Health Care Provider	Observational	Product/Behavior Awareness Pre-behavioral Behavioral
Lugada, E., Millar, D., Haskew, J., Grabowsky, M., Garg, N., Vestergaard, M., et al. (2010). Rapid implementation of an integrated large-scale HIV counseling and testing, malaria, and diarrhea prevention campaign in rural Kenya. PloS One, 5(8), e12435.	Rural	Sub-Saharan Africa	Male Condom	Promotion	Mass media Community Outreach Community Mobilization Health Care Provider	Observational	Behavioral
Martinez-Donate, A. P., Zellner, J. A., Fernandez-Cerdeno, A., Sanudo, F., Hovell, M. F., Sipan, C. L., et al. (2009). Hombres sanos: Exposure and response to a social marketing HIV prevention campaign targeting heterosexually identified Latino men who have sex with men and women. Aids Education and Prevention, 21,(5, B), 124-136}.	LGBTQ Men Minority	US	Male Condom	Place Promotion	Mass media Community Outreach	Observational	Product/Behavior Awareness Pre-behavioral Behavioral
McBride, S. D. (2003). A tailored minimal self-help intervention to promote condom use in young women: Results from a randomized trial. Aids, 17(10), 1547-1556.	Adolescents Women	US	Male Condom	Promotion	Mass media	Experimental	Pre-behavioral Behavioral

Citation	Populations	Region	Product	4 P(s) of Marketing	Intervention Components	Design	Significant Effects
McCool-Myers, M. (2019). Implementing condom distribution programs in the united states: Qualitative insights from program planners. Evaluation and Program Planning, 74, 20-26.	Adolescents	US	Both Male and Female Condom	Place Promotion	Mass media	Observational	Product/Behavior Awareness Sales and Distribution
Meekers, D. (2000). Going underground and going after women: Trends in sexual risk behaviour among gold miners in south Africa. International Journal of STD and AIDS, 11(1), 21-26.	Men Other	Sub-Saharan Africa	Male Condom	Promotion	Community Outreach	Observational	Product/Behavior Awareness Pre-behavioral Behavioral
Meekers, D. (2000). The effectiveness of targeted social marketing to promote adolescent reproductive health: The case of Soweto, South Africa. Journal of HIV/AIDS Prevention and Education for Adolescents and Children, 3(4), 73-92.	Adolescents Women	Sub-Saharan Africa	Male Condom	Product Price Place Promotion	Mass media Earned media Community Outreach Community Mobilization Health Care Provider	Quasi-Experimental	Pre-behavioral
Meekers, D., & Richter, K. (2005). Factors associated with use of the female condom in Zimbabwe. International Family Planning Perspectives, 31(1), 30-37.	Women Men Urban	Sub-Saharan Africa	Female Condom	Product Price Place Promotion	Mass media Earned media	Observational	
Meekers, D., Agha, S., & Klein, M. (2005). The impact on condom use of the "100% jeune" social marketing program in	Adolescents	Sub-Saharan Africa	Female Condom	Place Promotion	Mass media Community Outreach	Observational	Pre-behavioral Behavioral

Citation	Populations	Region	Product	4 P(s) of Marketing	Intervention Components	Design	Significant Effects
Cameroon. Journal of Adolescent Health, 36(6), 530.e12.					Community Mobilization		
	Women Sex Workers Rural	Sub-Saharan Africa	Male Condom	Product Price Place Promotion		Observational	Pre-behavioral Behavioral
	Men Sex Workers	Sub-Saharan Africa	Male Condom	Product Price Place Promotion		Observational	Behavioral
Morris, C. N., Morris, S. R., & Ferguson, A. G. (2009). Sexual behavior of female sex workers and access to condoms in Kenya and Uganda on the trans-Africa highway. Aids and Behavior}, 13}(5), 860-865}. doi:{10.1007/s10461-008-9431-z	Adolescents	Sub-Saharan Africa	Male Condom	Place	Mass media Community Outreach Community Mobilization	Observational	Behavioral
Piot, B., Mukherjee, A., Navin, D., Krishnan, N., Bhardwaj, A., Sharma, V., et al. (2010). Lot quality assurance sampling for monitoring coverage and quality of a targeted condom social marketing programme in traditional and non-traditional outlets in India. Sexually Transmitted Infections}, 86}(1), 56-61.	Adolescents Women	India	Male Condom	Place	Mass media	Observational	Sales and Distributio n
Plautz, A., & Meekers, D. (2007). Evaluation of the reach and impact of the 100% jeune youth social marketing program in Cameroon: Findings from three	Women Men	Sub-Saharan Africa	Male Condom	Product Place Promotion		Observational	Product/Be havior Awareness Pre-

Citation	Populations	Region	Product	4 P(s) of Marketing	Intervention Components	Design	Significant Effects
cross-sectional surveys. Reproductive Health, 4 doi:10.1186/1742-4755-4-1							behavioral Behavioral
Porto, M. P. (2007). Fighting AIDS among adolescent women: Effects of a public communication campaign in Brazil. Journal of Health Communication, 12(2), 121-132. doi:10.1080/10810730601150072	Adolescents Young Adult	Latin America	Male Condom	Promotion	Mass media Community Outreach Mobile Phone	Observational	Pre-behavioral
Potter, W., & de Villemeur, M. (2003). Clinical breakage, slippage and acceptability of a new commercial polyurethane condom: A randomized, controlled study. Contraception, 68}(1), 39-45	General Populations	Western Europe/ Canada	Male Condom	Product	Social Media	Experimental	Product/Behavior Awareness
Purdy, C. H. (2006). Fruity, fun and safe: Creating a youth condom brand in Indonesia. Reproductive Health Matters, 14(28), 127-134.	Sex Workers	South East Asia	Male Condom	Product Price Place Promotion	Community Outreach Health Care Provider	Observational	Product/Behavior Awareness Sales and Distribution
Purdy, C. H. (2011). Using the internet and social media to promote condom use in turkey. Reproductive Health Matters, 19(37), 157-165.	LGBTQ Men	Central Asia	Male Condom	Price Promotion	Community Outreach Health Care Provider	Observational	Sales and Distribution
Rachakulla, H. K., Kodavalla, V., Rajkumar, H., Prasad, S. P. V., Kallam, S., Goswami, P., et al. (2011). Condom use and prevalence of syphilis and HIV among	LGBTQ Urban Minority	India	Male Condom	Product Place Promotion	Mass media Community Outreach	Observational	Behavioral

Citation	Populations	Region	Product	4 P(s) of Marketing	Intervention Components	Design	Significant Effects
female sex workers in Andhra Pradesh, India - following a large-scale HIV prevention intervention. BMC Public Health, 11 (6)							
Ramanathan, S., Deshpande, S., Gautam, A., Pardeshi, D. B., Ramakrishnan, L., Goswami, P., et al. (2014). Increase in condom use and decline in prevalence of sexually transmitted infections among high-risk men who have sex with men and transgender persons in Maharashtra, India: Avahan, the India AIDS initiative. BMC Public Health, 14	Urban	India	Male Condom	Place Promotion	Mobile Phone Social Media	Observational	Behavioral
Renaud, T. C., Bocour, A., Irvine, M. K., Bernstein, K. T., Begier, E. M., Sepkowitz, K. A., et al. (2009). The free condom initiative: Promoting condom availability and use in New York City. Public Health Reports, 124(4), 481-489.	Women Urban Minority Other	US	Male Condom	Price Place		Observational	Product/Behavior Awareness Behavioral Sales and Distribution
Ridlo, I. A., & Zein, R. A. (2018). #CondomEmoji: Are urban Indonesians receptive to a social media-based campaign for safer sex? Health Education, 118(5), 386-401. doi:10.1108/HE-02-2018-0010	Influencers	South East Asia	Male Condom	Product Promotion	Community Outreach	Observational	Pre-behavioral

Citation	Populations	Region	Product	4 P(s) of Marketing	Intervention Components	Design	Significant Effects
Robinson, B. E., Uhl, G., Miner, M., Bockting, W. O., Scheltema, K. E., Rosser, B. R. S., et al. (2002). Evaluation of a sexual health approach to prevent HIV among low income, urban, primarily African American women: Results of a randomized controlled trial. AIDS Education and Prevention, 14(3), 81-96.	Adolescents Health Care providers	US	Both Male and Female Condom	Product Promotion	Community Outreach Health Care Provider	Experimental	
Rovniak, L. S., Hovell, M. E., Hofstetter, C. R., Blumberg, E. J., Sipan, C. L., Batista, M. F., et al. (2010). Engaging community businesses in human immunodeficiency virus prevention: A feasibility study. American Journal of Health Promotion}, 24}(5), 347-353}.	Women Sex Workers	US	Male Condom	Product Place	Community Outreach Community Mobilization Health Care Provider	Observational	
Ryder, H., Aspden, T., & Sheridan, J. (2015). The hawke's bay condom card scheme: A qualitative study of the views of service providers on increased, discreet access for youth to free condoms. The International Journal of Pharmacy Practice, 23(6), 381-389.	Adolescents Women Urban Minority	Oceania	Male Condom	Place Promotion	Health Care Provider	Observational	Pre-behavioral
Sharma, V., Saggurti, N., & Bharat, S (2015). Association between system reach and exposure to interventions and characteristics of mobile female sex workers in four high HIV prevalence states in India. Global Journal of Health Science, 7(4), 83-95.	Adolescents Women Men Sex workers	India	Male Condom	Product Price Place Promotion	Mass media	Observational	Product/Behavior Awareness

Citation	Populations	Region	Product	4 P(s) of Marketing	Intervention Components	Design	Significant Effects
Sales, J. M., Brown, J. L., Diclemente, R. J., & Rose, E. (2012). Exploring factors associated with nonchange in condom use behavior following participation in an STI/HIV prevention intervention for African-American adolescent females. AIDS Research and Treatment	Rural	US	Male Condom	Product Promotion	Mass media Health Care Provider	Experimental	Pre-behavioral
Scott-Sheldon, L., Glasford, D. E., Marsh, K. L., & Lust, S. A. (2006). Barriers to condom purchasing: Effects of product positioning on reactions to condoms. Social Science and Medicine, 63(11), 2755-2769.	Adolescents	US	Male Condom		Mass media	Experimental	Pre-behavioral Sales and Distributio n
Seidenfeld, D. (2014). An intervention to increase the condom supply in rural Zambia. Studies in Family Planning}, 45}(3), 379-387}.	General Populations Adolescents Young Adult Minority	Sub-Saharan Africa	Male Condom	Place	Mass media Community Outreach Community Mobilization Health Care Provider	Observational	Sales and Distributio n
Seoane Pascual, L. (2002). Qualitative assessment of a campaign promoting condom use among a teenage and young adult population in the community of Madrid, Spain. Revista Espanola De Salud Publica, 76(5), 509-516. Retrieved	Men Other	Western Europe/ Canada/	Male Condom	Price Place	Mass media Community Outreach	Observational	Pre-behavioral Sales and Distributio n

Citation	Populations	Region	Product	4 P(s) of Marketing	Intervention Components	Design	Significant Effects
Sewak, A., & Singh, G. (2012). Assessment of the Fiji-based condom social marketing (CSM) program. Sexuality and Culture, 16(4), 389-407. doi:10.1007/s12119-012-9128-3	General Populations	Oceania	Male Condom	Product Price Place Promotion	Mass media	Observational	Product/Behavior Awareness Pre-behavioral
Sharma, V., Saggurti, N., & Bharat, S. (2015). Health care coverage among long-distance truckers in India: An evaluation based on the Tanahashi model. HIV Aids-Research and Palliative Care}, 7}, 83-94}. doi:{10.2147/HIV.S76416	LGBTQ Men Influencers	India	Male Condom	Product Place Promotion	Community Outreach Community Mobilization Health Care Provider	Observational	Sales and Distribution
Sood, S., & Nambiar, D. (2006). Comparative cost-effectiveness of the components of a behavior change communication campaign on HIV/AIDS in North India. Journal of Health Communication, 11, 143-162.	General Populations	India	Male Condom	Product Price Place Promotion		Observational	Pre-behavioral Behavioral
Tan, J., Cai, R., Lu, Z., Cheng, J., de Vlas, S. J., & Richardus, J. H. (2013). Joint marketing as a framework for targeting men who have sex with men in China: A pilot intervention study. AIDS Education and Prevention, 25(2), 102-111. doi:10.1521/aeap.2013.25.2.102	Adolescents	East Asia	Male Condom	Product Place Promotion	Community Outreach	Quasi-Experimental	Pre-behavioral Behavioral
Terris-Prestholt, F., & Windmeijer, F. (2016). How to sell a condom? The impact of demand creation tools on male and female condom sales in resource limited	Men Other	Sub-Saharan Africa	Both Male and	Product Price Place Promotion		Observational	Sales and Distribution

Citation	Populations	Region	Product	4 P(s) of Marketing	Intervention Components	Design	Significant Effects
settings. Journal of Health Economics, 48, 107-120.			Female Condom				
Tomnay, J. E., & Hatch, B. (2013). Council-supported condom vending machines: Are they acceptable to rural communities? Sexual Health, 10(5), 465-	Other	Oceania	Male Condom	Place		Observational	Sales and Distributio n
Tran, B. R., Thomas, A. G., Vaida, F., Ditsela, M., Phetogo, R., Kelapile, D., et al. (2014). An intervention study examining the effects of condom wrapper graphics and scent on condom use in the Botswana Defense Force. AIDS Care - Psychological and Socio-Medical Aspects of AIDS/HIV, 26(7), 890-898.	Women Men	Sub-Saharan Africa	Male Condom	Product	Mass media Health Care Provider	Quasi-Experimental	Behavioral
Uhrig, J. D., Bann, C. M., Wasserman, J., Guenther-Grey, C., & EroÇ§lu, D. (2010). Audience reactions and receptivity to HIV prevention message concepts for people living with HIV. AIDS Education and Prevention, 22(2), 110-125.	General Populations	US	Male Condom	Promotion	Mass media Community Outreach Community Mobilization	Experimental	Pre-behavioral
Van, A. S. (2001). The impact of mass media campaigns on intentions to use the female condom in Tanzania Washington, D.C., Population Services International PSI], Research Division, 2001.	Adolescents	Sub-Saharan Africa	Female Condom	Product Price Place Promotion	Mass media Community Outreach Community Mobilization Health Care Provider	Observational	Pre-behavioral

Citation	Populations	Region	Product	4 P(s) of Marketing	Intervention Components	Design	Significant Effects
Van Rossem, R., & Meekers, D. (2000). An evaluation of the effectiveness of targeted social marketing to promote adolescent and young adult reproductive health in Cameroon. AIDS Education and Prevention, 12(5), 383-404.	Adolescents<br>Women<br>Men	Sub-Saharan Africa	Male Condom	Place<br>Promotion	Mass media	Quasi-Experimental	Pre-behavioral<br>Behavioral
Weigold, T. D. (2001). AIDS public service announcements: Effects of fear and repetition on predictors of condom use. Health Marketing Quarterly, 18(3-4), 39-61.	Adolescents	US	Male Condom	Promotion	Not Reported	Experimental	Pre-behavioral
Williams, J. L., Christensen, C. J., Cagle, H. H., & Homan, C. E. (2001). Brief report on the effect of providing single versus assorted brand name condoms to hospital patients: A descriptive study. BMC Public Health, 1, 1-4.	Indigenous	US	Male Condom	Product<br>Place	Not Reported	Quasi-Experimental	Sales and Distributio n
Young, T. M., Marks, M. J., Zaikman, Y., & Zeiber, J. A. (2017). Situational influences on condom purchasing. Sexuality and Culture, 21(4),	Women<br>Men	US	Male Condom	Place	Not Reported	Quasi-Experimental	Pre-behavioral
